# Reconstructive Surgery of the Female Genital, Urethral, and Anal Tract: A Multidisciplinary Review and Future Perspectives

**DOI:** 10.3390/jpm15120613

**Published:** 2025-12-08

**Authors:** Vivian Del Sorbo, Paola Pentangelo, Paolo Verrazzo, Ritapia Papa, Carmine Alfano

**Affiliations:** 1 Department of Obstetrics and Gynaecology, University of Naples “Federico II”, 80131 Naples, Italy; vivian.delsorbo@unina.it; 2 Plastic Surgery Unit, Department of Medicine, Surgery and Dentistry, University of Salerno, 84081 Baronissi, Italy; r.papa4@studenti.unisa.it (R.P.); calfano@unisa.it (C.A.); 3 Department of Obstetrics and Gynaecology, Santa Maria delle Grazie Hospital, 80078 Pozzuoli, Italy; paolo.verrazzo@aslnapoli2nord.it

**Keywords:** pelvic disorders, gynecology, reconstructive surgery, aesthetic surgery

## Abstract

**Background**: Pelvic floor dysfunctions, congenital anomalies, and acquired defects of the female genital, urethral, and anal tract represent complex conditions requiring multidisciplinary management. This review synthesizes current evidence and aims to evaluate reconstructive surgical techniques, prosthetic use, and cosmetic approaches with a focus on functional and aesthetic outcomes. **Methods**: A structured literature search of PubMed, Embase, and the Cochrane Library was performed for the period from January 2000 to May 2025 (last search: 31 May 2025). Eligible studies included randomized controlled trials, prospective or retrospective clinical studies, and case series in English or Italian, enrolling women aged ≥18 years with a minimum follow-up of 6 months. Primary outcomes were anatomical restoration, continence, sexual function, and quality of life; secondary outcomes included patient satisfaction, wound complications, donor-site morbidity, and recurrence. Recent high-quality studies published after 2018 were prioritized to ensure an updated and evidence-based synthesis. **Results**: Out of 532 records, 94 full texts were assessed and 41 studies met the eligibility criteria, comprising a total of 1862 women. Flap-based reconstruction (gluteus maximus, VRAM, gracilis, ALT) remained the cornerstone for large or irradiated defects, while prosthetic meshes improved anatomical outcomes but raised concerns of erosion and chronic pain, leading to a shift toward autologous tissue. Cosmetic gynecology procedures (labiaplasty, vaginoplasty, perineoplasty) showed high patient satisfaction, although the evidence was limited and heterogeneous. Across studies, improvements were observed in continence, sexual function, quality of life, and self-image when reconstructive and aesthetic principles were integrated. **Conclusions**: Reconstructive and cosmetic pelvic surgery significantly impacts functional recovery and psychological well-being. Standardized outcome reporting, prospective multicenter trials, and the integration of plastic surgery, physiotherapy, and psychological support are needed to optimize patient-centered care. The findings highlight the growing role of personalized surgical planning, with reconstructive decisions increasingly guided by patient-specific anatomy, functional goals, and validated patient-reported outcome measures.

## 1. Introduction

Pelvic floor dysfunctions and disorders of the female genital, urethral, and anal tract constitute a group of conditions with profound implications for continence, sexual health, and quality of life. Congenital malformations such as vaginal agenesis or cloacal anomalies, as well as acquired defects following childbirth, oncologic surgery, or complex obstetric fistulas, may severely compromise functional and psychosocial well-being, particularly when extensive pelvic exenteration or complex reconstructive procedures are required [1,2].

Over the last decades, reconstructive techniques have evolved to include both pedicled and microsurgical flaps, prosthetic devices, and cosmetic approaches [3,4,5]. These strategies have expanded therapeutic options, but challenges remain regarding long-term efficacy, complication rates, and standardization of surgical outcomes [6,7]. Recent advances (2019–2025) have introduced refinements in perforator-based flaps, biologic grafts, and patient-specific reconstructive planning, significantly influencing both functional and aesthetic outcomes.

Recent years have also witnessed growing demand for gynecological cosmetic surgery, reflecting not only functional needs but also patients’ expectations for improved aesthetics and self-perception [8,9]. This trend has reinforced the importance of adopting a multidisciplinary approach that integrates gynecology, plastic surgery, physiotherapy, and psychological support [10,11,12].

In line with the principles of personalized medicine, modern pelvic reconstructive surgery places increasing emphasis on patient-specific factors, including anatomical variability, tissue quality, comorbidities, and individualized functional and aesthetic expectations. This paradigm shift is supported by novel imaging tools, preoperative planning algorithms, and standardized patient-reported outcome measures.

Although several systematic and narrative reviews have examined selected components of pelvic and perineal reconstruction, existing literature generally focuses on either oncologic reconstruction, obstetric trauma, or cosmetic procedures in isolation. To our knowledge, few reviews have attempted to integrate functional, aesthetic, oncologic, and congenital reconstructive strategies into a unified multidisciplinary framework. By combining microsurgical, gynecologic, urologic, and aesthetic perspectives, the present review fills a methodological gap and provides a comprehensive synthesis of reconstructive options across diverse indications.

Recent high-level evidence published in 2024–2025 has further expanded reconstructive options, particularly with refinements in perforator-based flap microsurgery, robotic-assisted pelvic reconstruction, and the integration of imaging-guided preoperative planning tools.

The objective of this review is to synthesize current evidence on reconstructive and aesthetic surgical approaches to the female uro-anogenital tract, evaluate their functional and aesthetic outcomes, and identify future perspectives for multidisciplinary, patient-centered care.

## 2. Methods

### 2.1. Search Strategy

A comprehensive literature search was performed in PubMed, Embase, and the Cochrane Library for articles published between 1 January 2000 and 31 May 2025 (last search: 31 May 2025). Search terms included: “pelvic floor reconstruction,” “female genital reconstruction,” “urogenital flaps,” “prosthetic mesh,” and “aesthetic gynecology.” Reference lists of included articles were also screened manually for additional eligible studies. The review was conducted following the PRISMA Statement guidelines [1].

The review protocol was preregistered on the Open Science Framework (OSF; DOI: https://doi.org/10.17605/OSF.IO/A5MWS) and PROSPERO (ID:1239372). The OSF registration has been made publicly accessible and includes full search strings, version history, predefined screening criteria, and data extraction templates to ensure full methodological transparency. The PRISMA checklist is provided in the Supplementary Table S1.

Full search strategies for PubMed, Embase, Cochrane and Scopus—detailing Boolean operators, MeSH terms, and date/language filters—are provided in Supplementary Table S2 [2].

In line with current publication standards, studies published after 2018 were given analytical priority to ensure contemporary relevance.

### 2.2. Inclusion and Exclusion Criteria

Studies were eligible if they met the following criteria:

- Design: randomized controlled trials, prospective or retrospective clinical studies, and case series.
- Population: women aged ≥18 years.
- Setting: hospital or tertiary care centers.
- Follow-up: minimum of 6 months.
- Interventions: reconstructive or aesthetic procedures involving the pelvic floor, perineum, genital, urethral, or anal tract.
- Outcomes: anatomical restoration, continence, sexual function, quality of life, patient satisfaction, wound complications, donor-site morbidity, or recurrence.
- Languages: English or Italian.

In addition, existing systematic reviews were screened to identify further eligible primary studies, but they were not themselves included in the qualitative synthesis.

Exclusion criteria were:

- Single case reports.
- Conference abstracts without full text.
- Studies lacking postoperative outcomes data relevant to the predefined endpoints.
- Animal or cadaveric studies.
- Publications in languages other than English or Italian.

Detailed inclusion and exclusion criteria are summarized in Supplementary Table S3.

### 2.3. Study Selection Process

A total of 532 records were initially identified. After removal of 138 duplicates, 394 titles and abstracts were screened, of which 300 were excluded for irrelevance. Ninety-four full-text articles were assessed for eligibility, and 41 studies were included in the final synthesis.

### 2.4. Bias Minimization Strategies

To reduce risk of bias, two independent reviewers screened titles/abstracts and assessed full texts. Discrepancies were resolved by a third senior reviewer. Data extraction was conducted using a standardized template.

- For observational studies, the Newcastle–Ottawa Scale (NOS) was applied [3].
- For randomized controlled trials, the Cochrane Risk of Bias (RoB 2.0) tool was used [2].

### 2.5. PRISMA Flowchart

The study selection process is summarized as follows:

- Identification: 532 records retrieved → 138 duplicates removed → 394 screened.
- Screening: 394 records screened → 300 excluded.
- Eligibility: 94 full-text articles assessed → 53 excluded.
- Included: 41 studies in the final qualitative synthesis.

The PRISMA 2020 flow diagram is provided in Supplementary Figure S1, in accordance with PRISMA guidelines.

### 2.6. Anatomical and Pathophysiological Considerations for Reconstructive Surgery

A precise understanding of female pelvic anatomy and pathophysiology is essential for evaluating reconstructive outcomes and guiding surgical strategies. The pelvic floor consists of three muscular layers: the deep layer (levator ani complex: pubococcygeus, puborectalis, iliococcygeus, plus coccygeus muscle), the intermediate layer (urogenital diaphragm), and the superficial layer (bulbocavernosus, ischiocavernosus, superficial transverse perineal muscles). These structures are interconnected by the endopelvic fascia, which transmits forces to the ligaments and provides suspension of the bladder, uterus, vagina, and rectum [4,13,14].

From a functional perspective, the pelvic floor ensures urinary and fecal continence, pelvic organ support, and sexual function. Damage to these structures may occur due to obstetric trauma, aging, menopause, pelvic surgery, or connective tissue disorders, resulting in conditions such as pelvic organ prolapse, stress or urgency urinary incontinence, fecal incontinence, and sexual dysfunction [15,16].

The vagina and urethra share connective, vascular, and innervating structures, explaining why surgical interventions targeting one compartment frequently influence the other. Similarly, the anal sphincter complex (internal and external sphincters) and the perineal body are pivotal for continence and sexual function, but are highly vulnerable to obstetric lacerations and oncological resections [17,18].

Accessory glands, including Bartholin’s and Skene’s glands, play roles in lubrication and protection of the lower genital tract; their preservation during reconstructive procedures is recommended to optimize postoperative comfort and reduce risk of fistula or abscess formation [19].

This anatomical and pathophysiological framework provides the rationale for reconstructive surgery: restoring anatomy not only re-establishes structural integrity, but also addresses continence, sexual function, and patient self-perception, which are central to long-term quality of life.

#### 2.6.1. The Pelvic Floor

The pelvic floor is a complex musculo-fascial structure that plays a crucial role in female pelvic statics, providing support to the pelvic organs (bladder, uterus, and rectum), ensuring urinary and fecal continence, and contributing to sexual function. From an anatomical point of view, it is conventionally divided into three main layers, each of which has specific biomechanical and clinical functions relevant in the gynecological field.

Deep Layer

Consisting of the levator ani muscle—divided into pubococcygeus, puborectalis, and iliococcygeus —and the ischiococcygeus muscle, it forms the so-called pelvic diaphragm. This layer represents the key element in supporting the pelvic organs and maintaining continence. The puborectalis muscle, in particular, plays a central role in maintaining the anorectal angle, while the pubococcygeus and iliococcygeus contribute to the overall integrity of the pelvic compartment.

Intermediate Layer

Corresponds to the urogenital diaphragm, mainly composed of the deep transverse perineal muscle and the middle perineal fascia. This muscular plane stabilizes the urethra and vagina, cooperating with the deep layer in preventing urogenital prolapse and modulating urinary continence, especially during increases in intra-abdominal pressure.

Superficial Layer

Includes the bulbocavernosus, ischiocavernosus, and superficial transverse perineal muscles. Although these muscles are less involved in the direct support of pelvic organs, they play a significant role in sexual function and in stabilizing the perineal body, acting synergistically with the upper layers to maintain perineal integrity.

Endopelvic Fascia

A fundamental structural element, the endopelvic fascia connects the muscles of the pelvic floor to the walls of the pelvic organs and supporting structures (e.g., cardinal and uterosacral ligaments), contributing to the suspension and proper positioning of the organs within the pelvis.

Clinical Implications in Gynecology

Pelvic floor dysfunctions are among the main causes of gynecological morbidity, significantly impacting patients’ quality of life. The primary etiopathogenic factors include obstetric trauma, aging, pelvic surgeries, menopause, and genetic predisposition. Clinical manifestations include pelvic organ prolapse, stress or urgency urinary incontinence, fecal incontinence, and sexual dysfunctions.

A thorough understanding of the surgical anatomy of the pelvic floor, integrated with the pathophysiology of its dysfunctions, is essential for accurate clinical assessment and for the development of targeted therapeutic strategies—both conservative and surgical—within modern gynecology.

#### 2.6.2. Vagina

The vagina is a fibromuscular canal extending from the vulvar vestibule to the cervix, with an average length of 7–10 cm. It is lined by a non-keratinized stratified squamous epithelium and lacks endogenous glands. Lubrication is provided by transudate from the vaginal wall and cervical secretions. From a histological point of view, the vaginal wall is composed of three layers: mucosa, muscular, and adventitia. The mucosa has transverse folds (rugae) that allow expansion during childbirth and sexual intercourse. The smooth muscle is organized into an inner circular layer and an outer longitudinal layer, while the adventitia contains connective tissue rich in elastic fibers and blood vessels.

#### 2.6.3. Urethra

The female urethra is a short conduit (about 3–5 cm) extending from the bladder to the external urethral meatus, located in the vulvar vestibule. It is lined by transitional epithelium in the proximal portion and by stratified squamous epithelium in the distal portion. The urethral wall is composed of an inner layer of smooth muscle and an outer layer of striated muscle, which contributes to the voluntary control of urination. The urethra is closely connected to the anterior vaginal wall and surrounded by erectile tissue, including the bulbs of the vestibule and the Skene’s glands.

#### 2.6.4. Accessory Glands

Skene’s glands (paraurethral glands): Located laterally to the urethra, these glands are homologous to the male prostate and secrete a fluid that contributes to urethral lubrication and may be involved in female ejaculation. Histologically, they consist of tubuloalveolar structures lined by columnar or cuboidal epithelium and surrounded by connective tissue and smooth muscle.

Bartholin’s glands (greater vestibular glands): Located bilaterally at the vaginal vestibule, in a postero-lateral position relative to the vaginal orifice, these glands secrete mucus to lubricate the vaginal introitus. The excretory ducts open into the vestibule around 4 and 8 o’clock positions. They are homologous to the male bulbourethral (Cowper’s) glands.

Clinical relevance Surgical procedures involving the Skene’s and Bartholin’s glands must account for the potential impact on lubrication and the risk of postoperative complications such as fistula formation or abscesses. Glandular preservation, when feasible, is often advisable during reconstructive procedures.

#### 2.6.5. Anus

The anus is the terminal opening of the anal canal, located below the vagina. It is surrounded by two sphincters: internal (involuntary smooth muscle) and external (voluntary striated muscle). The external sphincter is further divided into subcutaneous, superficial, and deep parts. The puborectalis muscle, part of the levator ani muscle, wraps around the anal canal posteriorly, contributing to the maintenance of fecal continence.

### 2.7. Outcomes

The primary outcomes were:

1. Anatomical restoration—closure or reconstruction confirmed by clinical or imaging follow-up.
2. Continence—urinary continence (absence of stress/urgency incontinence) and fecal continence (absence of soiling/urgency), measured by validated questionnaires (e.g., ICIQ-UI, Wexner score) or clinical evaluation.
3. Sexual function—improvement or preservation assessed by validated instruments (e.g., FSFI) or patient-reported outcomes.
4. Quality of life—measured by standardized tools (e.g., SF-36, EQ-5D, disease-specific instruments).

The secondary outcomes included:

- Patient satisfaction and self-image,
- Wound complications (infection, dehiscence, necrosis, flap loss),
- Donor-site morbidity,
- Reoperation rates and recurrence of defects (prolapse, fistula, incontinence).

For all outcomes, a minimum follow-up of 6 months was required for eligibility, and data were extracted at the longest reported follow-up.

### 2.8. Risk of Bias Assessment

Two reviewers independently assessed risk of bias. Disagreements were resolved by consensus with a third senior reviewer.

Randomized trials (RoB 2.0). We used the Cochrane Risk of Bias 2.0 tool across the five domains: (1) randomization process; (2) deviations from intended interventions; (3) missing outcome data; (4) measurement of the outcome; (5) selection of the reported result. Each domain and the overall study were rated as Low risk, Some concerns, or High risk, supported by verbatim quotes or page references from the article.

Observational studies (NOS). We applied the Newcastle–Ottawa Scale (Selection/Comparability/Outcome or Exposure, max 9 stars). Following a priori decision rules, overall quality categories were mapped as: Low risk (≥7 stars), Some concerns (5–6 stars), High risk (≤4 stars).

Calibration and agreement. Before formal assessment, reviewers calibrated judgments on three pilot studies. Inter-rater agreement (Cohen’s κ) was calculated and reported in Supplementary Figure S1.

How risk of bias informed the synthesis. We prespecified that:

- Primary narrative conclusions prioritize Low risk studies;
- Studies at High risk contribute to context but do not drive conclusions;
- We performed sensitivity analyses excluding High risk evidence and highlighting any changes in direction/strength of effects;
- When results conflicted, we applied narrative weighting favoring lower-risk, larger, and more directly applicable studies.

Per-study tables are presented in Supplementary Table S4.

### 2.9. Data Synthesis Rules

Given the heterogeneity of study designs and outcomes, we adopted a structured narrative synthesis. A priori decision rules were defined to group studies into clinically meaningful categories:

- By anatomical defect: pelvic floor/perineal, vaginal, urethral, anal, or combined.
- By reconstructive technique: pedicled flaps, microsurgical free flaps, perforator flaps, prosthetic meshes, or cosmetic/aesthetic procedures.
- By clinical context: oncologic resections, obstetric/traumatic injuries, congenital anomalies, or elective cosmetic indications.
- By tissue status: irradiated versus non-irradiated fields.
- By surgical setting: high-resource (tertiary university hospitals) versus lower-resource environments.

Within each category, evidence was synthesized narratively, with emphasis on primary outcomes (anatomical restoration, continence, sexual function, quality of life). When overlapping indications or techniques were reported, studies were classified according to the predominant indication or reconstructive method.

## 3. Results

A total of 532 records were identified through database searching. After removal of duplicates and screening, 41 studies met the eligibility criteria and were included in the final synthesis. The study selection process is summarized in the PRISMA flow diagram (Supplementary Figure S1), including reasons for full-text exclusions.

Studies were grouped according to predefined decision rules (see Methods), including defect type, reconstructive technique, clinical context, tissue status (irradiated vs. non-irradiated), and surgical setting. Given the pronounced heterogeneity in indications (oncologic, obstetric, congenital, cosmetic) and techniques (pedicled flaps, perforator flaps, biologic grafts, synthetic meshes), we stratified the evidence by both indication and procedural category. This allows clearer interpretation of outcomes and direct comparison of reconstructive versus aesthetic procedures. This approach ensured consistency and comparability across heterogeneous designs and outcomes.

### 3.1. Outcome of Risk of Bias Assessment

Risk of bias varied across the included studies. Among randomized controlled trials, most were rated as low risk or with some concerns in the domains of randomization and outcome measurement, while missing outcome data frequently introduced concerns. Observational studies generally achieved 5–7/9 stars on the Newcastle–Ottawa Scale, corresponding to some concerns. Only a minority were judged at high risk of bias. Sensitivity analyses excluding high-risk studies did not materially change the direction or strength of the main findings; therefore, conclusions are primarily based on low-risk evidence, with high-risk studies reported for contextual value. Detailed per-study assessments, supporting quotes, and traffic-light figures are provided in Supplementary Table S3 and Figure S1.

### 3.2. Pelvic Floor and Perineal Reconstruction

Several studies emphasized personalized flap selection based on defect geometry, prior irradiation, body habitus, and functional rehabilitation requirements.

The included studies described a wide range of reconstructive options for pelvic floor and perineal defects, ranging from local fasciocutaneous flaps to microsurgical techniques.

The gluteus maximus myocutaneous flap was consistently reported as a workhorse for large sacral and perineal defects. It incorporates both muscle and overlying skin, with vascular supply from the inferior and superior gluteal arteries. Several series emphasized its use in irradiated or complex wounds, where its robust vascularization supports healing and reduces risk of breakdown [4,5].

The vertical rectus abdominis myocutaneous (VRAM) flap, harvested vertically from the rectus abdominis muscle and supplied by the deep inferior epigastric vessels, was highlighted as a first-choice option for deep pelvic reconstruction following exenteration or complex fistula repair. Evidence indicates reliable cavity obliteration and improved wound healing, although donor-site morbidity remains a limitation [6,20].

For medium-sized defects, the gracilis muscle flap was frequently reported. Its dominant vascular pedicle from the medial femoral circumflex artery allows safe mobilization, and its pliability makes it suitable for perineal, vaginal, or urethral reconstructions. Clinical outcomes showed good functional recovery, particularly in irradiated fields or recurrent fistula repair [7,8].

The anterolateral thigh (ALT) flap, based on the lateral circumflex femoral artery perforators, was another versatile option for large or complex pelvic and perineal defects. It may be harvested as fasciocutaneous, myocutaneous, or with fascia lata, providing large surface coverage and adequate bulk with relatively low donor-site morbidity [21].

For inguinal and perineal regions requiring structural support, the tensor fascia lata (TFL) flap was reported as particularly useful due to its mechanical strength and large skin paddle, although bulkiness may be a limitation [22].

In sacral and ischial regions, superior and inferior gluteal artery perforator (SGAP/IGAP) flaps were described as reliable alternatives, especially when muscle preservation was desirable. These perforator-based flaps provided good coverage while sparing gluteal function, which is crucial for rehabilitation in ambulatory patients [4,23].

Finally, for superficial perineal defects, smaller local fasciocutaneous flaps such as the pudendal thigh flap were associated with good cosmetic outcomes and low morbidity. They were often indicated in non-irradiated, superficial defects where muscle coverage was not required [7].

Overall, flap selection depended on defect size, depth, location, history of irradiation, and availability of microsurgical expertise. While most studies reported high rates of anatomical restoration, complications included wound dehiscence, partial flap necrosis, fistula recurrence, and donor-site morbidity. Variability in outcome measures across studies limited direct comparison, underscoring the need for standardized reporting in future research [4,5,6,7,8,20,21,22,23].

### 3.3. Vaginal and Urethral Reconstruction

The studies addressing vaginal and urethral reconstruction emphasized the importance of restoring both anatomy and function, particularly continence and sexual health.

Vaginal reconstruction outcomes were significantly influenced by personalized choice of tissue (colon, fasciocutaneous flap, gracilis), tailored to patient age, sexual activity, lubrication needs, and coexisting pelvic floor disorders.

For vaginal reconstruction, one of the most widely reported options was the sigmoid colon flap (sigmoid colpoplasty). A pedicled segment of sigmoid colon, harvested with its vascular pedicle, was mobilized to create a neovagina. This technique was especially used in congenital anomalies such as Mayer–Rokitansky–Küster–Hauser (MRKH) syndrome and in post-oncological or traumatic defects. Clinical reports highlighted excellent depth, lubrication, and long-term stability of the neovagina, although complications such as mucus hypersecretion or stenosis were occasionally described [5,6].

Another frequently described technique was the pudendal thigh flap, which uses fasciocutaneous tissue based on branches of the external pudendal artery. This flap was reported as a reliable option for partial or circumferential vaginal defects, providing good elasticity and satisfactory cosmetic outcomes with low donor-site morbidity [7]. Similarly, the gracilis flap and gluteal flaps were described in extensive or irradiated defects, offering robust vascularized coverage when fasciocutaneous options were insufficient [7,8].

In the context of urethral reconstruction, the Martius flap was one of the most frequently reported techniques. This procedure mobilizes fatty and muscular tissue from the labia majora, based on the internal pudendal artery. It is tunneled under the vaginal wall and positioned to reinforce the urethral repair. The Martius flap was consistently reported as effective in the treatment of urethro-vaginal fistulas, providing vascularized tissue that enhances healing and reduces recurrence rates [19].

Other described approaches for urethral repair included the use of skin grafts, inguinal flaps, and transverse myocutaneous gracilis flaps, particularly in complex or recurrent cases. While functional continence rates were generally satisfactory, heterogeneity in patient populations and small sample sizes limited comparability [7,8].

Overall, evidence indicates that vaginal and urethral reconstruction benefits from tissue vascularity, elasticity, and functional integration. Flap choice should be tailored according to the extent of tissue loss, history of radiotherapy, and patient-specific needs for continence and sexual function.

### 3.4. Use of Prostheses in Pelvic Surgery

Several studies reported on the use of prosthetic materials in pelvic reconstructive surgery, particularly in the management of stress urinary incontinence (SUI) and pelvic organ prolapse (POP).

For SUI, mid-urethral slings (MUS) using polypropylene mesh remain the most widely adopted technique. Positioned tension-free under the mid-urethra, these slings provide structural support during increases in intra-abdominal pressure. Long-term studies with follow-up exceeding 10 years have demonstrated cure or improvement rates above 80% [9]. However, mesh-related complications such as erosion, chronic pain, and persistent or de novo urgency symptoms were reported, with exposure rates ranging from 5 to 10%. Risk factors for mesh exposure included smoking, prior pelvic surgery, and poor tissue quality [9].

For vaginal vault prolapse after hysterectomy, abdominal sacrocolpopexy (ASC) with mesh was frequently described as the “gold standard” technique. This procedure involves fixation of the vaginal vault to the sacral promontory using a synthetic mesh. Prospective studies reported anatomic success rates between 90 and 100% for apical prolapse, with improvements in urinary and sexual function [10]. However, mesh erosion occurred in up to 8–21% of patients, and some series documented persistent anorectal dysfunction despite good anatomic correction [10].

Comparisons between laparoscopic sacrocolpopexy (LSC) and vaginal mesh repairs suggested higher anatomical success and lower reoperation rates for LSC, supporting the superiority of minimally invasive abdominal approaches [10].

Beyond synthetic prostheses, several reports evaluated the use of biological meshes, derived from autologous fascia lata, rectus fascia, or xenografts of porcine/bovine origin. These materials demonstrated lower erosion rates and better integration but were associated with higher recurrence of prolapse compared to synthetic mesh.

Recent publications emphasized regulatory concerns. The U.S. Food and Drug Administration (FDA) and the European Medicines Agency (EMA) issued warnings regarding the risk of complications after transvaginal mesh implantation, recommending cautious patient selection and informed consent. Consequently, native tissue repair and autologous slings are increasingly considered safe alternatives in selected populations.

Overall, prosthetic use in pelvic reconstruction provides high anatomical success rates, but long-term safety concerns and regulatory restrictions highlight the need for individualized treatment planning and preference for native tissue when feasible.

### 3.5. Post-Traumatic Perineal Repair and Reconstruction

Several studies focused on perineal injuries resulting from obstetric trauma or surgical complications, highlighting both immediate repair strategies and complex secondary reconstructions.

Obstetric perineal tears are classified into four degrees, ranging from superficial mucosal involvement to complete disruption of the anal sphincter and rectal mucosa. Evidence indicates that continuous suturing techniques may reduce operative time and material use compared with interrupted sutures, without differences in pain or healing outcomes [16]. For third- and fourth-degree tears, the overlapping sphincter repair technique was associated with lower rates of anal incontinence compared with end-to-end repair, underlining the importance of sphincter restoration in functional outcomes [17].

In cases of complex post-traumatic perineal disruption, including injuries from surgical complications, sexual assault, or severe accidents, more advanced reconstructive procedures have been reported. The posterior sagittal anorectoplasty (PSARP), initially developed for congenital anorectal malformations, has been adapted for post-traumatic cases. This approach enables precise separation of rectum and vagina, reconstruction of the perineal body, and restoration of the anal sphincter complex. Case series reported satisfactory continence and cosmetic results following this technique [18].

Postoperative management was emphasized as a determinant of long-term outcome. Strategies included careful wound care, pain control, physiotherapy, and scheduled follow-up to assess sphincter function and detect complications such as dyspareunia, infection, or persistent pain [16,19].

Overall, evidence highlights that accurate classification of the injury, selection of the appropriate suturing or reconstructive technique, and structured postoperative care are crucial to preserve perineal function and quality of life after trauma.

### 3.6. Obstetric Fistula Repair

Obstetric fistulas, most commonly vesicovaginal and rectovaginal fistulas, remain a severe complication of obstructed labor, particularly in low-resource settings. The studies included in this review emphasized both traditional and innovative reconstructive strategies aimed at restoring continence and vaginal function.

The Martius flap, mobilizing fatty and muscular tissue from the labia majora, was one of the most frequently reported approaches for small or recurrent fistulas. Its use provided well-vascularized tissue interposition, improving healing rates and reducing recurrence [19].

For more complex or large fistulas, the rectus abdominis muscle flap was described as an effective option. A case series from sub-Saharan Africa reported restoration of continence in the majority of patients within three months postoperatively, with minimal donor-site morbidity [6]. Similarly, the Singapore fasciocutaneous flap, based on pudendal vessels and transposed from the inguinal region, was employed in cases complicated by vaginal stenosis or scarring, offering reliable tissue coverage and improved sexual function [7].

Emerging techniques such as autologous fat grafting for recurrent anovaginal fistulas demonstrated promising results, providing a minimally invasive option with reduced dissection and low donor-site morbidity. Early outcomes suggested high rates of symptom improvement, though larger studies are needed to confirm long-term efficacy [8].

Predictors of successful repair included fistula size, absence of circumferential urethral involvement, and limited vaginal scarring. Conversely, complex or irradiated fistulas and those with urethral destruction were associated with lower success rates [6,7,8].

In low-resource settings, simple flaps such as the gracilis and Singapore flap were particularly valued for their reliability and limited requirement for advanced infrastructure. Several studies emphasized the importance of surgeon expertise and treatment in specialized centers, reporting higher repair success and improved functional outcomes when procedures were performed in high-volume units [7].

### 3.7. Cosmetic Gynecology Procedures

Over the past two decades, there has been a steady rise in the demand for cosmetic gynecology procedures, often performed for both functional improvement and enhancement of body image or sexual satisfaction. Cosmetic procedures increasingly rely on individualized planning, guided by anatomical mapping, sexual function goals, and patient-reported outcome instruments such as the validated Vulvovaginal Aesthetic Satisfaction Scale. The studies included in this review reported several techniques, although the overall evidence remains heterogeneous and limited by small sample sizes and lack of standardized outcomes.

Perineoplasty was commonly described in women with obstetric trauma, wide genital hiatus, or perineal laxity. The procedure involves excision of redundant tissue and approximation of superficial perineal muscles. Case series demonstrated improvement in vaginal tightness, sexual satisfaction, and relief from dyspareunia [11].

Vaginoplasty, performed to narrow the vaginal canal, was reported both after childbirth and in women with subjective complaints of laxity. Techniques included dissection and plication of the levator ani muscles, with or without mucosal excision. While many women reported enhanced sexual function, studies highlighted the absence of high-quality randomized trials, and outcomes remain largely patient-reported [12].

Labiaplasty was the most frequently reported cosmetic procedure. Techniques included edge resection, wedge resection, and de-epithelialization. According to classification systems proposed by Ellsworth and González, surgical choice was tailored to hypertrophy type and symmetry. Across multiple reports, labiaplasty showed high satisfaction rates (>90%), with low complication rates, though risks of scarring and altered sensation remain.

Clitoral hood reduction (hoodoplasty) was often performed in combination with labiaplasty, aiming to improve clitoral exposure and sexual function. While generally safe, its benefits are based on patient perception rather than standardized measures.

Other reported procedures included hymenoplasty, usually performed for cultural or personal reasons, and monsplasty for reduction in excess adipose tissue in the mons pubis. Both procedures demonstrated satisfactory aesthetic outcomes, but evidence is limited to case reports and small case series.

Finally, G-spot augmentation with autologous fat or dermal fillers has been described as an investigational procedure. Available evidence is anecdotal and inconsistent, with risks of granuloma formation or inflammatory reactions. Current consensus suggests this procedure should be considered experimental until more robust data are available [13].

Overall, cosmetic gynecology procedures are associated with high levels of patient satisfaction, but the absence of long-term data and objective functional assessments underscores the need for prospective studies with standardized outcomes.

Across the 41 included studies, the median sample size was 42 patients (IQR 18–67), with a median follow-up of 18 months. Reported anatomical success ranged between 82 and 100% for flap reconstruction and between 65 and 93% for prosthetic or minimally invasive repairs. Patient-reported satisfaction in cosmetic procedures consistently exceeded 85%, although follow-up was typically shorter (6–12 months).

To improve clarity and structural consistency, the main findings are summarized in two tables: Table 1, which differentiates functional reconstructive procedures from aesthetic procedures, and Table 2, which details sample characteristics, interventions, and primary outcomes for each included study.

## 4. Discussion

This structured review highlights the breadth of reconstructive and cosmetic surgical strategies for the female pelvic floor and uro-anogenital tract.

Our updated synthesis, which incorporates evidence from 2019 to 2025, confirms the rapid evolution of flap design, biologic grafts, and minimally invasive reconstructive strategies. These contemporary findings reinforce the shift toward techniques prioritizing functionality, aesthetics, and patient-specific anatomical variability. Across the included studies, several consistent themes emerged: from a clinical perspective, flap selection should be guided by defect location (perineal vs. vaginal vs. sacral), defect depth, tissue quality, and history of radiotherapy. VRAM flaps remain most reliable for deep oncologic cavities; gracilis flaps are preferred for moderate defects or fistulas; perforator flaps are advantageous when muscle preservation is desired. In contrast, synthetic meshes are reserved for selected cases with preserved tissue quality due to regulatory and complication-related constraints.

Within reconstructive pelvic surgery, flap-based approaches continue to represent the pivotal foundation for managing extensive defects, particularly in irradiated, infected, or previously operated fields where reliable vascularized tissue is essential. Pedicled and microsurgical flaps—including gluteus maximus, VRAM, gracilis, and ALT—provide robust coverage, durable healing, and low donor-site morbidity, and remain the standard of care for large oncologic or traumatic pelvic resections [4,5,6,7,8,20,21,22,23].

Prosthetic use remains effective but controversial, as biologic and synthetic meshes can restore anatomical support and reduce surgical time, yet carry persistent concerns regarding erosion, chronic pain, and re-intervention, particularly in cases of prior radiotherapy or compromised tissue quality. The recent shift toward mesh-free or hybrid reconstructive strategies reflects the need to balance anatomical restoration with long-term safety and patient-reported outcomes [9,10].

Post-traumatic pelvic and perineal repair requires tailored, context-specific strategies, as injuries related to high-energy trauma or complex soft-tissue loss often involve multilevel disruption of the pelvic floor and perineal body. These cases benefit from early involvement of reconstructive surgeons and from individualized decision-making that integrates tissue viability, contamination, and expected functional sequelae, especially in continence and sexual function [16,17,18,19].

Obstetric fistula repair remains one of the most challenging areas of pelvic reconstruction, particularly in settings where tissue fibrosis, repeated failed repairs, or extensive perineal disruption coexist. Flap-based interposition—such as the gracilis or VRAM flap—offers improved healing and reduced recurrence by re-establishing vascularized support between the urinary, genital, or anorectal tracts. Nevertheless, evidence remains heterogeneous, and long-term functional outcomes continue to be underreported [6,7,8].

Cosmetic gynecology is expanding but remains under-researched, with increasing patient demand for procedures addressing labial hypertrophy, vaginal laxity, and aesthetic dissatisfaction. Despite high satisfaction rates, the literature is limited by small samples, heterogeneous techniques, and inconsistent outcome reporting. Standardization and validated patient-reported outcome measures are essential to ensure ethical and evidence-based adoption of these procedures [11,12,13].

Multidisciplinary approaches remain a central component of optimal care, as complex pelvic reconstruction benefits from coordinated expertise involving gynecology, urology, colorectal surgery, plastic and reconstructive surgery, physiotherapy, radiology, and psychological support. Integrating functional goals, aesthetic expectations, and quality-of-life considerations within shared decision-making frameworks is crucial to achieving durable outcomes and patient-centered success [13,14,15].

A major emerging theme across recent literature is the transition toward personalized reconstructive and aesthetic surgery. Patient-specific factors—such as tissue elasticity, prior surgeries, comorbidities, sexual function goals, continence profile, and aesthetic expectations—are increasingly used to guide flap selection, mesh indication, and cosmetic procedures. The integration of patient-reported outcomes and shared decision-making frameworks further strengthens the application of precision medicine in uro-genital and pelvic floor reconstruction.

## 5. Conclusions

Reconstructive and cosmetic surgery of the female genital, urethral, and anal tract has advanced considerably, offering a wide range of techniques tailored to congenital anomalies, acquired trauma, oncologic resections, and functional or aesthetic needs. Evidence consistently supports flap-based reconstruction as the cornerstone for complex defects, while the use of prosthetic materials remains effective but controversial due to long-term safety concerns.

Post-traumatic perineal repair and obstetric fistula management highlight the importance of surgical expertise, multidisciplinary collaboration, and structured postoperative care. Cosmetic gynecology procedures demonstrate high patient satisfaction, yet robust data on long-term functional outcomes are lacking.

Future research should prioritize standardized outcome reporting, the development of core outcome sets for both functional and aesthetic procedures, and prospective multicenter trials and registries capturing long-term anatomical, functional, and patient-reported outcomes. Personalized surgical algorithms integrating functional anatomy, advanced imaging, and validated patient-reported outcome measures are needed to refine indication criteria and optimize reconstruction. Multidisciplinary pathways that combine gynecology, plastic surgery, physiotherapy, and psychological support will be crucial to maximize continence, sexual function, and overall quality of life in women undergoing pelvic reconstructive surgery.

## Figures and Tables

**Table 1:** Summary of functional reconstructive procedures and aesthetic procedures, including indications, techniques, follow-up, primary outcomes, and complications profiles across the included studies.

Category	Main Indications	Typical Techniques	Median Sample Size (Range)	Typical Follow-Up	Primary Outcomes	Complication Profile
Functional Reconstructive Procedures	Oncologic resections; obstetric trauma; congenital anomalies; fistulas; irradiated fields	VRAM flap; Gracilis flap; ALT flap; SGAP/IGAP; Martius flap; Sigmoid colpoplasty; Autologous slings	30–60 patients (range 12–150)	12–36 months	Anatomical restoration; continence (urinary/fecal); sexual function; QoL	Wound dehiscence (5–15%); donor-site morbidity; flap necrosis (1–5%); recurrence (5–20%)
Aesthetic (Cosmetic) Procedures	Labial hypertrophy; vaginal laxity; perineal laxity; aesthetic dissatisfaction; clitoral hood redundancy	Labiaplasty (edge, wedge, de-epithelialization); Vaginoplasty; Perineoplasty; Hoodoplasty; Monsplasty	20–45 patients (range 10–80)	6–12 months	Self-reported sexual satisfaction; body image; aesthetic satisfaction	Minor wound dehiscence (<5%); altered sensation (1–3%); asymmetry (3–5%); scar-related complaints (2–7%)

Abbreviations: ALT, anterolateral thigh; SGAP, superior gluteal artery perforator; IGAP, inferior gluteal artery perforator; VRAM, vertical rectus abdominis myocutaneous; QoL, quality of life.

**Table 2:** Characteristics, indications, interventions, follow-up length, and primary outcomes of key studies included in the review.

Study (Year)	Population (N, Setting)	Indication	Intervention/Technique	Follow-Up	Primary Outcomes
Chen et al., 2022 [4]	58 women, tertiary center	Pelvic oncologic defects	MS-VRAM flap	24 months	Anatomical restoration, wound healing
PelvEx Collaborative, 2024 [5]	112 patients, multicenter	Pelvic exenteration	Multimodal flap reconstruction	36 months	QoL, functional recovery
Patel et al., 2025 [6]	14 women, LMIC	Complex obstetric fistula	Rectus abdominis flap	12 months	Continence, fistula closure
Pope et al., 2018 [7]	21 women	Vaginal stenosis post-fistula	Singapore flap	18 months	Sexual function, anatomical patency
Braga et al., 2022 [9]	137 SUI patients	Stress urinary incontinence	TVT-Abbrevo	60 months	Continence rates, pain
van Oudheusden et al., 2023 [10]	74 women	Vault prolapse	Laparoscopic sacrocolpopexy	72 months	Recurrence, anatomical success
Ozbasli et al., 2022 [12]	60 women	Benign indications	Robotic vs. laparoscopic cosmetic procedures	12 months	Aesthetic satisfaction
Arnold et al., 2021 [17]	90 perineal tears	Obstetric trauma	Overlapping vs. end-to-end repair	12 months	Anal continence outcomes
White & Atchan, 2022 [19]	55 postpartum	Perineal injury	Standard repair + structured management	6 months	Pain, healing, dyspareunia

Abbreviations: LMIC, low- and middle-income countries; SUI, stress urinary incontinence; TVT, tension-free vaginal tape; QoL, quality of life; MS-VRAM, muscle-sparing vertical rectus abdominis myocutaneous.

## Data Availability

No new data were created or analyzed in this study. Data sharing is not applicable to this article.

## Supplementary Materials

The following supporting information can be downloaded at: https://www.mdpi.com/article/10.3390/jpm15120613/s1, Table S1: The PRISMA checklist; Table S2: Search strategies; Table S3: Detailed inclusion and exclusion criteria; Table S4: Per-study tables; Figure S1: PRISMA 2020 flow diagram of study selection for the review.
