# Supplementary material for: Reconstructive Surgery of the Female Genital, Urethral, and Anal Tract: A Multidisciplinary Review and Future Perspectives
Source: J Pers Med. 2025 Dec 8;15(12):613. doi: 10.3390/jpm15120613 (PMC12734212; doi:10.3390/jpm15120613)
Supplement: Supplementary file 1 [file jpm-15-00613-s001.zip › Supplementary table S2 - Search strategies.pdf]

Database	Search strategy
**PubMed (MEDLINE)**	("pelvic floor reconstruction"[MeSH Terms] OR "pelvic floor"[All Fields] OR "perineal reconstruction"[All Fields]) OR ("female genital reconstruction"[All Fields] OR "urogenital flaps"[All Fields]) OR ("prosthetic mesh"[MeSH Terms] OR "mesh"[All Fields]) OR ("aesthetic gynecology"[All Fields] OR "cosmetic gynecology"[All Fields]) AND ("surgery"[MeSH Terms] OR "surgical procedures, operative"[All Fields]) AND ("2000/01/01"[Date - Publication] : "2025/05/31"[Date - Publication]) AND (English[lang] OR Italian[lang])
**Embase (Ovid)**	('pelvic floor reconstruction'/exp OR 'perineum reconstruction':ti,ab,kw) OR ('female genital reconstruction':ti,ab,kw OR 'urogenital flap':ti,ab,kw) OR ('prosthetic mesh'/exp OR 'surgical mesh':ti,ab,kw) OR ('aesthetic gynecology':ti,ab,kw OR 'cosmetic gynecology':ti,ab,kw) AND ('surgery'/exp OR 'surgical procedure':ti,ab,kw) AND [2000-2025]/py AND (english:la OR italian:la)
**Cochrane Library (CENTRAL)**	(pelvic floor reconstruction):ti,ab,kw OR (perineal reconstruction):ti,ab,kw OR (female genital reconstruction):ti,ab,kw OR (urogenital flap):ti,ab,kw OR (prosthetic mesh):ti,ab,kw OR (aesthetic gynecology):ti,ab,kw OR (cosmetic gynecology):ti,ab,kw AND (surgery):ti,ab,kw Publication Year from 2000 to 2025 Language: English OR Italian
