# Supplementary material for: Reconstructive Surgery of the Female Genital, Urethral, and Anal Tract: A Multidisciplinary Review and Future Perspectives
Source: J Pers Med. 2025 Dec 8;15(12):613. doi: 10.3390/jpm15120613 (PMC12734212; doi:10.3390/jpm15120613)
Supplement: Supplementary file 1 [file jpm-15-00613-s001.zip › Supplementary table S3 - Detailed inclusion and exclusion criteria.pdf]

Domain	Inclusion criteria	Exclusion criteria
**Study design**	Randomized controlled trials (RCTs), clinical studies, case series, systematic reviews	Single case reports; conference abstracts without full text
**Population**	Women aged ≥18 years	Patients <18 years
**Setting**	Hospital or tertiary care centers	Non-clinical or experimental-only settings
**Follow-up**	Minimum 6 months	Studies without follow-up or <6 months
**Language**	English, Italian	Publications in other languages
**Intervention**	Reconstructive or aesthetic surgery of the pelvic floor, perineum, genital, urethral, or anal tract	Studies not involving surgical reconstruction
**Outcomes**	Anatomical restoration; continence; sexual function; quality of life; patient satisfaction; wound complications; donor-site morbidity; recurrence	Studies lacking outcome data
