# Supplementary figures and images for: Reconstructive Surgery of the Female Genital, Urethral, and Anal Tract: A Multidisciplinary Review and Future Perspectives
Source: J Pers Med. 2025 Dec 8;15(12):613. doi: 10.3390/jpm15120613 (PMC12734212; doi:10.3390/jpm15120613)

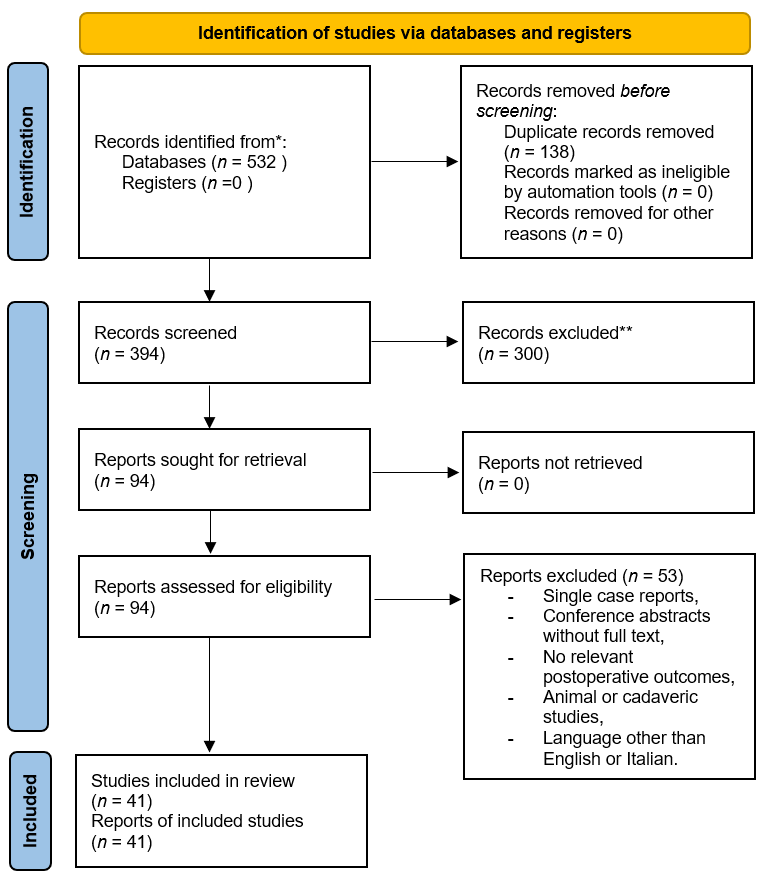

Supplement: Supplementary file 1 [file jpm-15-00613-s001.zip › Supplementary Figure S1 - PRISMA 2020 flow diagram of study selection for the review.png]
